# Advanced backward planning with custom-milled individual allogeneic block augmentation for maxillary full-arch osteoplasty and dental implantation:a 3-year follow-up

**DOI:** 10.1007/s10561-021-09947-3

**Published:** 2021-08-09

**Authors:** Manfred Nilius, Charlotte Mueller, Minou Helene Nilius, Dominik Haim, Bernhard Weiland, Guenter Lauer

**Affiliations:** 1 Niliusklinik, Londoner Bogen 6, 44269 Dortmund, Germany; 2 Department of Oral and Maxillofacial Surgery, University Hospital “Carl Gustav Carus”, Technische Universität Dresden, Fetscherstr. 74, 01307 Dresden, Germany

**Keywords:** Allogeneic, Alveolar atrophy, Augmentation, Dental implantation, Grafting, Maxilla

## Abstract

In the case of maxillary involution, augmentation is necessary for implant-supported prosthetics. The use of bone grafts is standard; customized allogeneic bone blocks may be a predictable alternative before dental implantation. For maxillary full-arch reconstruction, this case shows a horse-shoe augmentation by four allogeneic blocks, followed by guided dental implantation and fixed prosthetics after 6 months of healing. Using allogeneic blocks is an option for full-arch maxillary augmentation and comparable with autologous bone grafts. There is no donor site comorbidity. Bone height is stable for a minimum of 3 years after loading with resorption less than 10% in vertical, buccolingual, and mesiodistal directions. Short-implants allow for the long-term stability of prosthetic fixtures. Prefabricated customized allogeneic blocks for augmentation may increase the fitting accuracy of the graft, decrease morbidity, and lower operation time in maxillary full-arch reconstruction. The percentage of resorption after 3 years is comparable to the commonly used iliac crest.

## Introduction

No adequate amount of bone in the maxilla or a retromaxillary involution indicates bone block grafting or sinus lifting before implantation. Autologous transplants as gold standard bear comparable high stress for the patient (Cricchio and Lundgren 2003). In these cases, allogenic bone blocks can be a suitable alternative. In this article, customized allogeneic bone blocks are prepared for onlay-augmentation to improve bone height and bone quantity before dental implantation. After osseous integration of the blocks, advanced backward planned dental implants were the method of choice for a stable, secure fixing of prosthetic restoration.

**Fig. 1:**
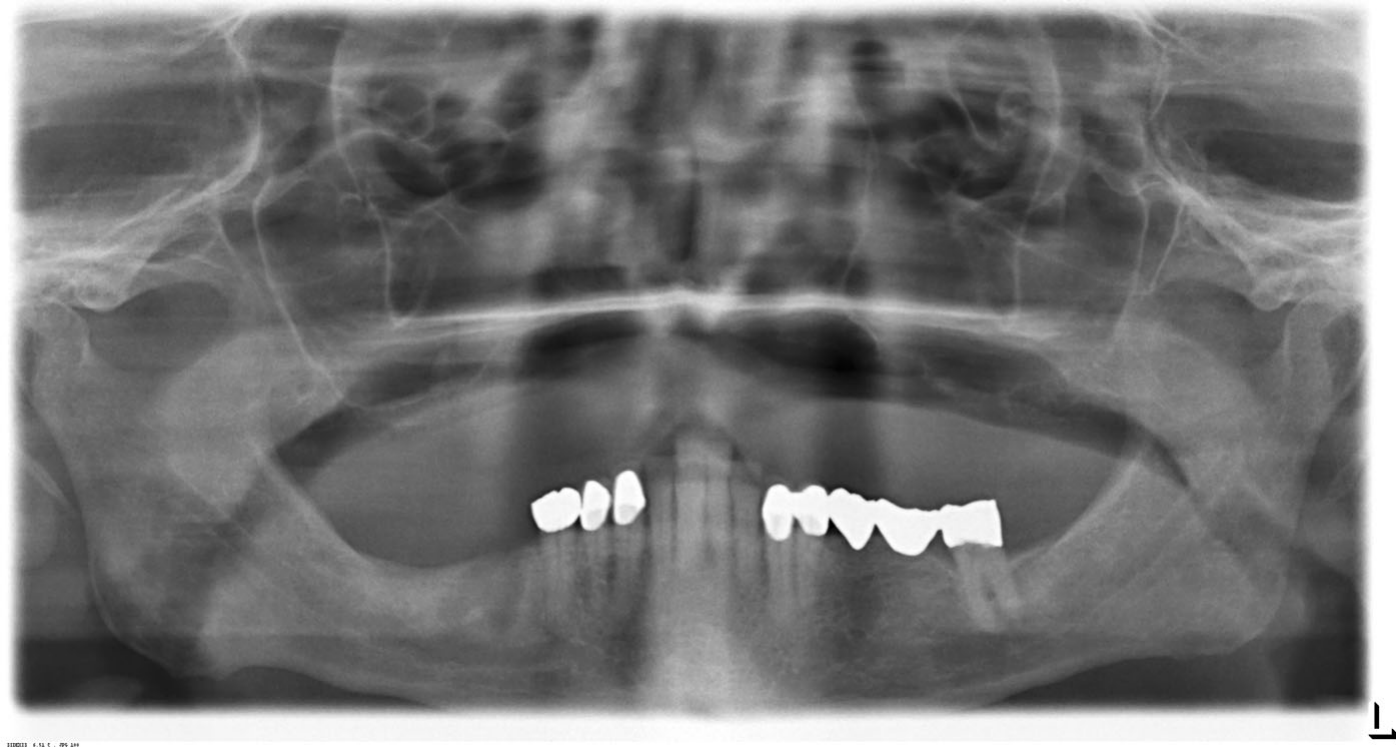
Preoperative OPT of a 72 y old male showing highly alveolar ridge atrophy in the maxilla before treatment

**Fig. 2:**
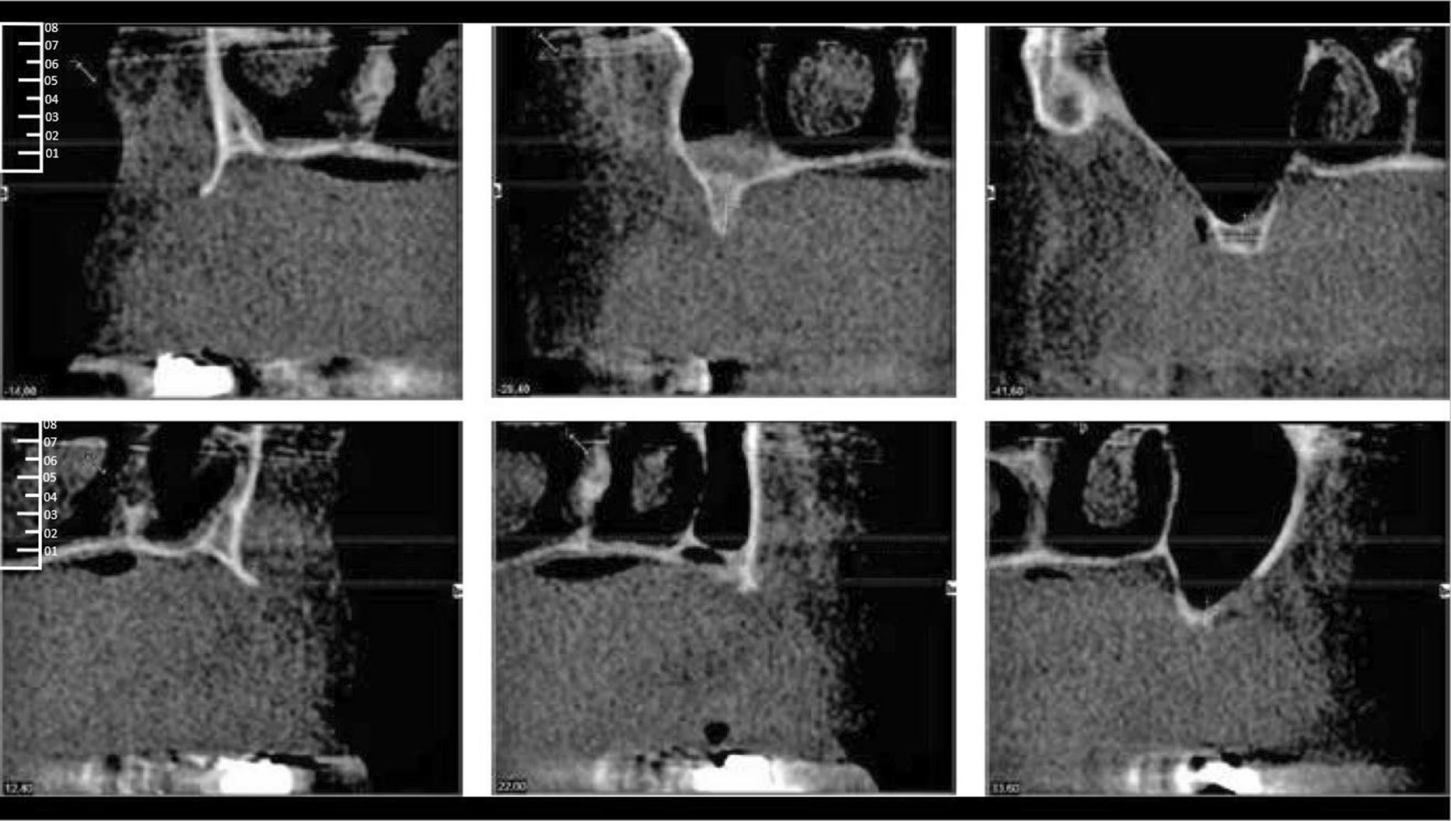
Transversal CBCT-Slices of the maxillary bone dimension before augmentation (region: 012, 015, 017 upper rows, 022, 024, 026 lower row)

In the case presented, a 72-year old patient with a small amount of residual bone height (vertical height ≤ 3 mm) in the edentulous maxilla consulted the clinic for an implant treatment (Figs. 1 and 2). The treatment need was a progredient insufficiency of the patient’s prosthesis, which mostly became evident as a cornetist in an orchestra. The high-grade alveolar ridge atrophy, the low palatal arch, and the edentulous upper jaw's retro position did not allow implantation into the local bone. Even an oblique insertion of the posterior implants into the local bone was impossible without extensive bone augmentation. The patient rejected zygoma-implants because palatal placed prosthetic abutments could narrow the tongue’s space; Le-Fort-1-Osteotomy or maxillary advancement with iliac crest declined because of operative load and possible complications at the donor site. Finally, maxillary augmentation by using customized allogeneic bone blocks was accepted.

## Method

### Advanced backward planning

**Fig. 3:**
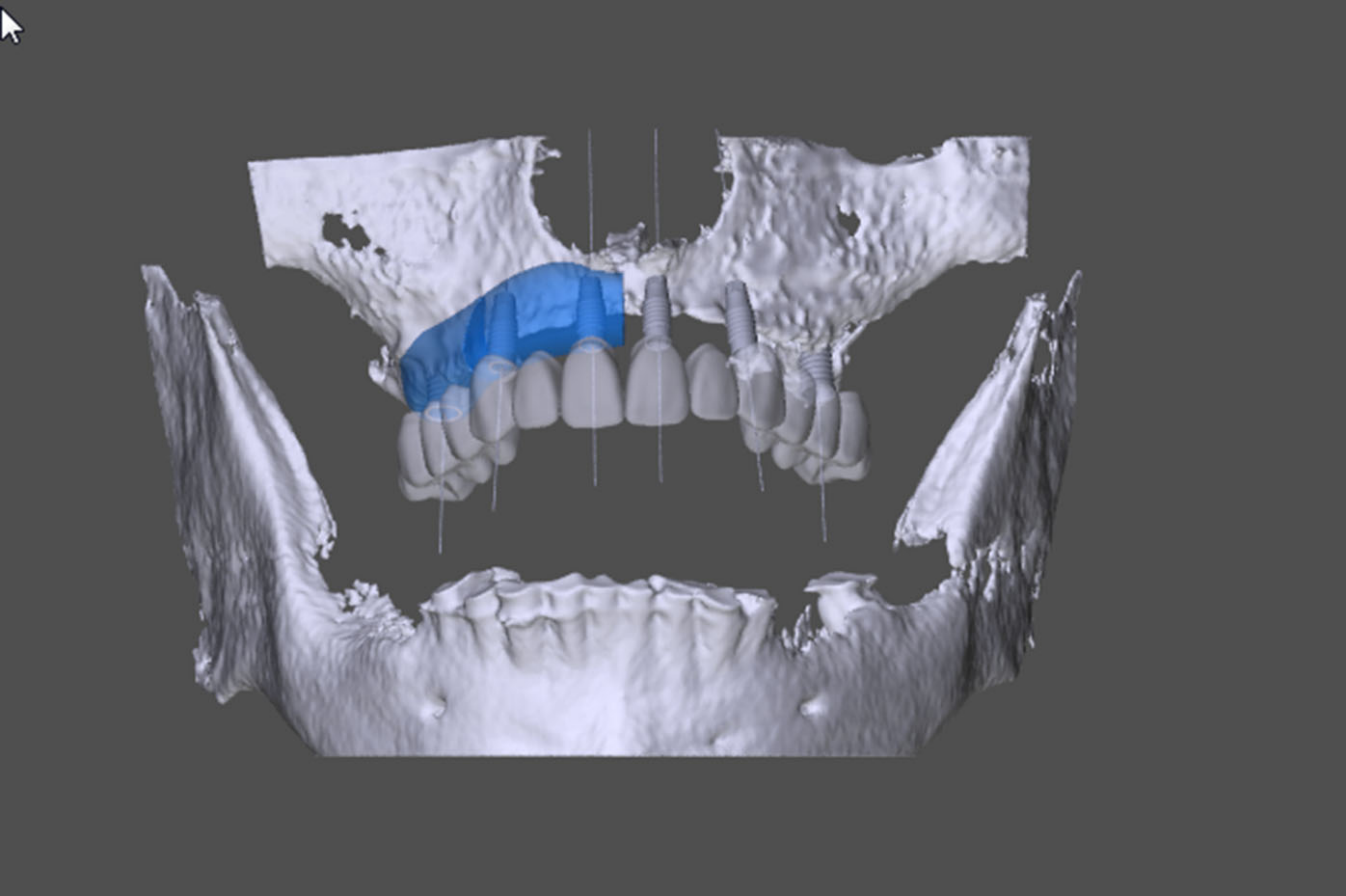
A virtual model of a CBCT for advanced backward planning of prosthetics and dental implants (grey) and customized milled Bonebuilder® for the maxilla (Blue; Botiss®-Software; Botiss-Straumann, CH-Basel). Anterior view

**Fig. 4:**
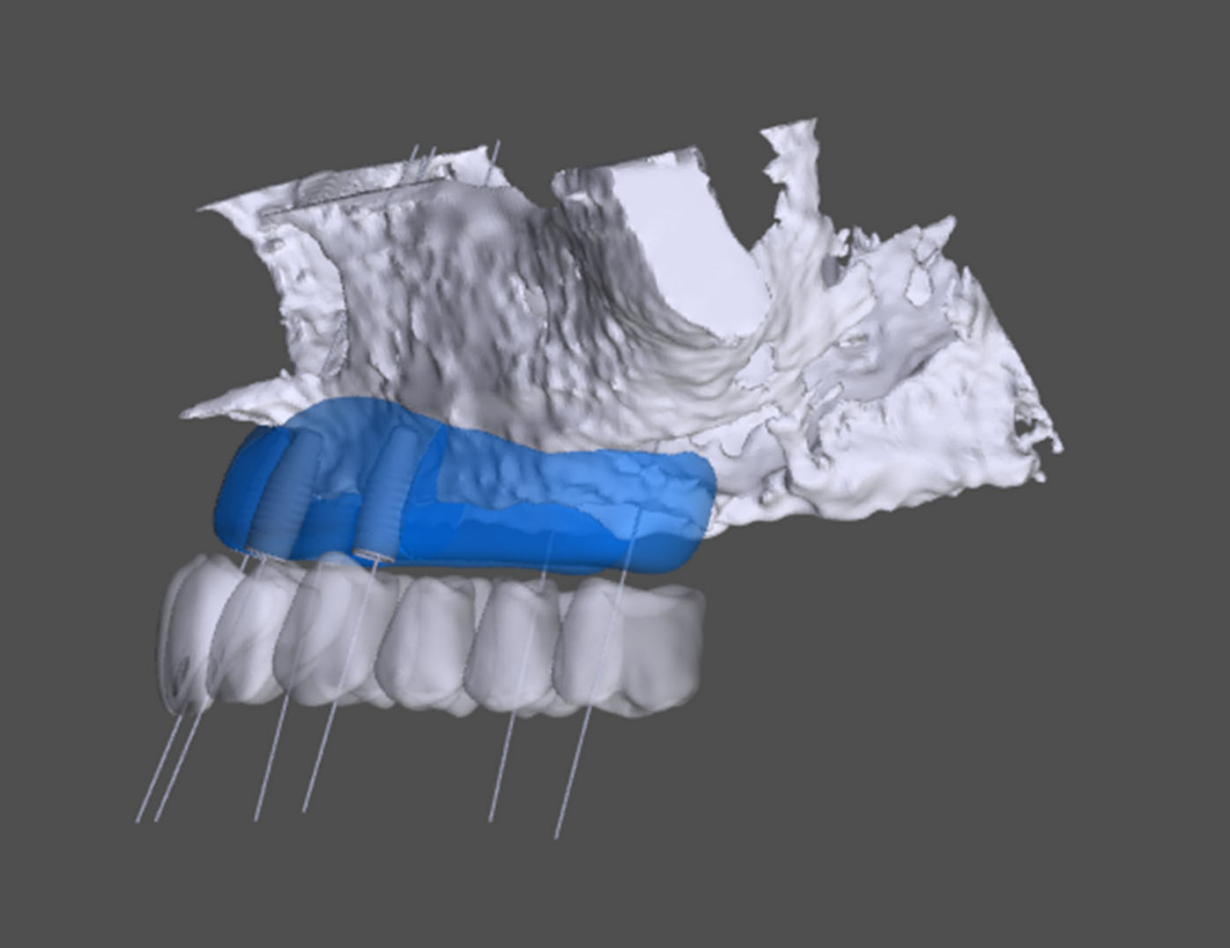
Virtual model of a CBCT for advanced backward planning for prosthetics and dental implants (grey) and customized milled Bonebuilder® for the maxilla (Blue; Botiss®-Software; Botiss-Straumann (CH-Basel). Lateral view

An advanced backward planning procedure is necessary to supply the atrophied maxillary bone with an allogeneic, customer-specific bone block. Based on a digital prosthetic mock-up, we anticipated the correct implant positions and the block size needed to equalize the bone loss. After CBCT, DICOM-data was exported and edited by the Botiss-CAD-Designer (Botiss, Berlin, Germany); (Figs. 3 and 4), we designed exactly fitting transplants on a virtual 3D-model in the shape of a horse-shoe. As there is a maximum size of the maxgraft bonebuilder of 23 × 13 × 13mm, we divided the horse-shoe into four single blocks. After the surgeon's approval, the blocks were milled out of a processed, allogeneic cancellous bone block under cleanroom conditions and, double-packed, got sterilized with gamma irradiation. The production and delivery times amount to approximately 5 weeks.

CBCT-scans were taken immediately after the grafting surgery, 6 months postoperatively at implant placement, and 36 months after functional loading of the implants. An initial CBCT scan anticipated grafting procedure for advanced-backward-planning and surgical purposes to investigate maxillary bone atrophy and dental implantation. We used the CBCT (Kavo-3D-Exam, Biberach, Germany; Software KaVoExamVision Version 1.9.3.12) with exposure factors of 120 kV and 36.12 mAs with a 0.1-mm reconstruction interval. We used heads of mini-screws as reference points; later, we used the implants themselves to evaluate vertical height, bucco-palatal, and mesiodistal distance. The initial value was compared with the dimensions after augmentation, after insertion, 6 months after implantation, 24 and 36 months after implantation. The bone dimensions were compared in terms of their total bone loss to initial bone formation by CBCT-measuring.

All surgeries were performed by the same experienced surgeon, according to the technique described above.

### Surgery

The patients maintained strict oral hygiene in the 2 weeks that preceded both grafting and surgical implant procedures.

Extensive mobilization of the soft tissue, a precise incision, and appropriate surgical suturing technique are crucial for a success-promising operation. We exposed the local bone through a vestibular, marginal incision. The authors recommend rehydrating the allogeneic bone in a disposables syringe with sterile isotonic saline solution (implant venting) to improve the transit of immigration acellular matrix via osmosis (Nilius et al. 2019). The periosteum of the palatal gingiva reached up to the median palatal raphe. We screwed each block with two mini-screws (1.2 × 10 mm, Stoma; MS Dental AG, CH-Busswill) to the palatal bone. The screws were placed angularly into the transplant or local bone, particularly crucial with the thin basal bone (crisscross-technique). Ideally, a screw position follows the future implant axis, which makes the movement relatively easier.

**Fig. 5:**
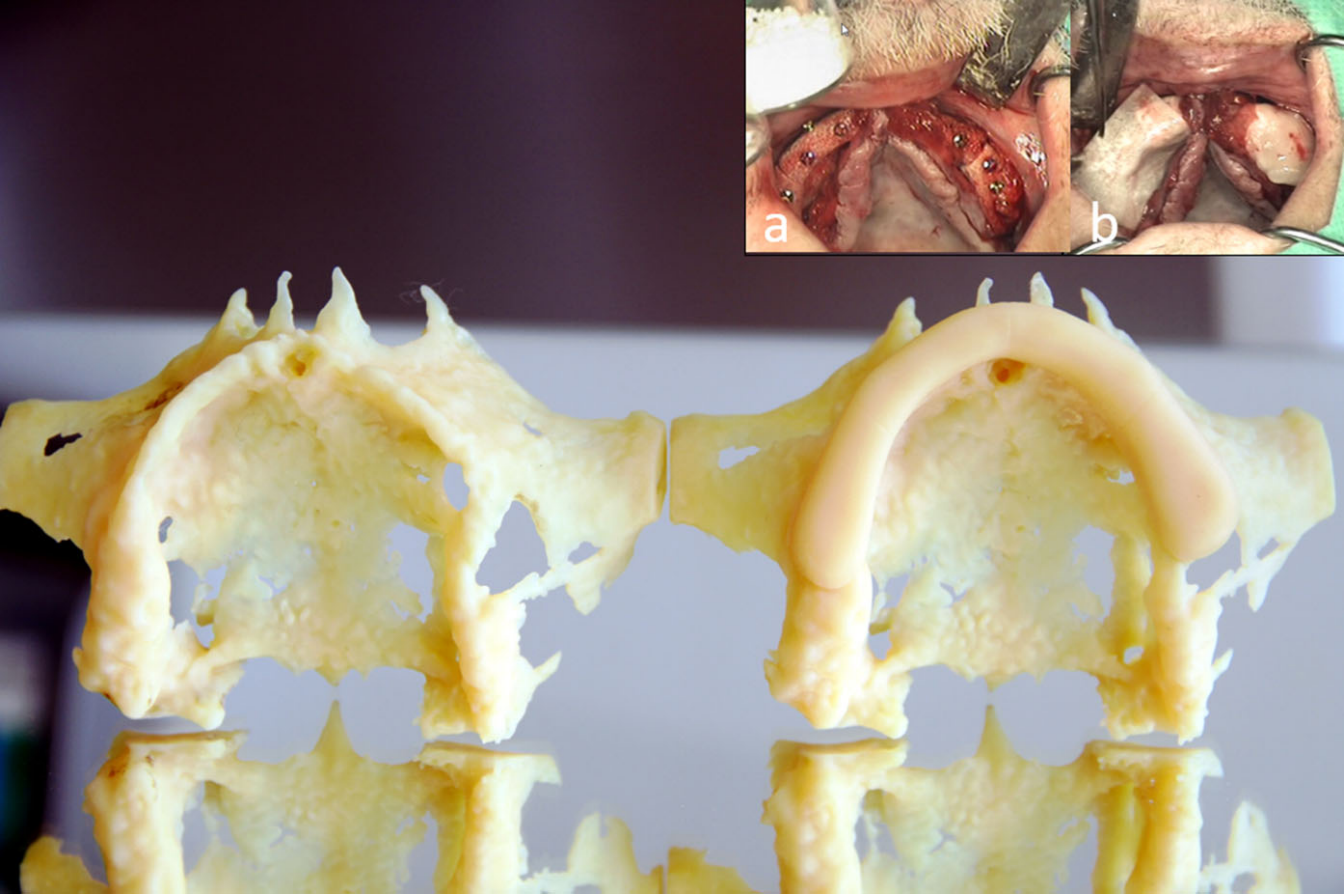
Picture from sterile Bonebuilder®-Dummies before (right) and after surgery (left): Details (**a**, **b**) from Video-Screenshot: **a** Inserting the bone blocks and rigid fixation with mini-screws, and grinding of allogeneic bone particles (glass-bottom) to smooth out superficial irregularities; **b** Membranous protection of the bone blocks with PRGF coverage

**Fig. 6:**
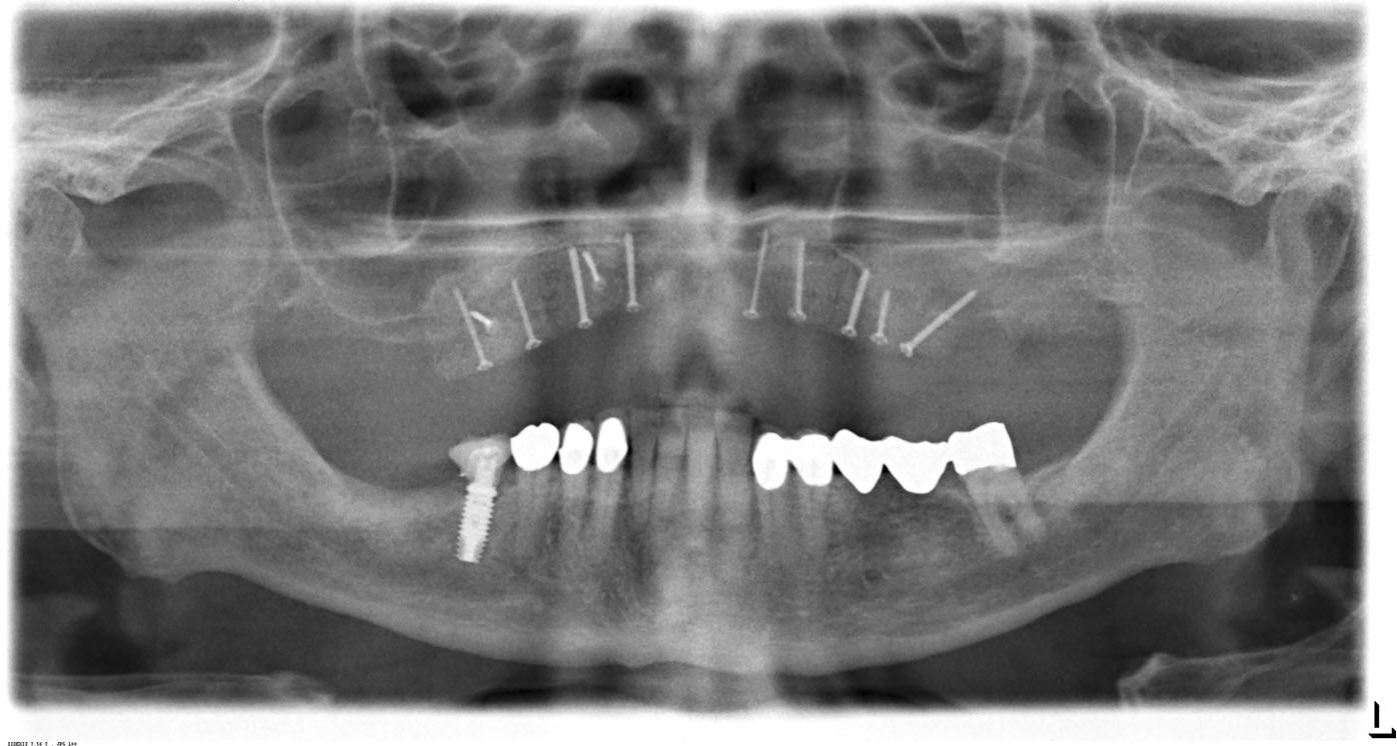
OPT after screw fixation of the allogeneic bone blocks in the maxilla

**Fig. 7:**
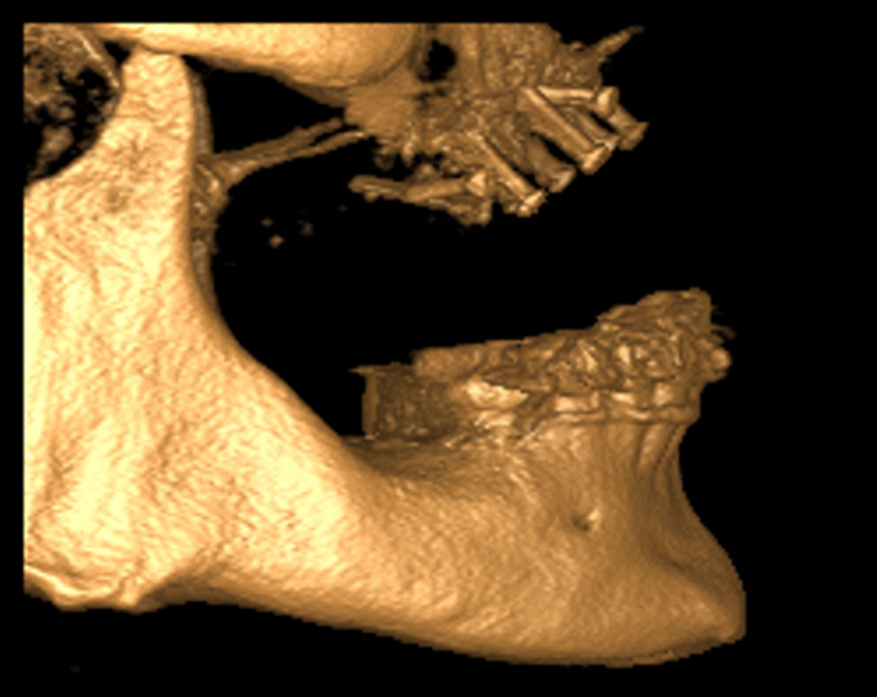
CBCT (3D- rendered; 3DVR, KaVo,) Lateral view before dental implantation and screw-removement

The bone segments need coverage entirely by a barrier membrane (Jason-membrane, Botiss, Straumann, CH-Basel) or PRGF as described by Anitua et al. (2009) or both in combination. To prevent fast proliferating fibroblasts and epithelial cells migration into the bone block and keep space for controlled bone regeneration. The mucoperiosteal flap was sutured saliva-proof and without tension (Figs. 5, 6 and 7). We performed no additional sinus grafting. The patient was highly compliant and was not wearing any prosthesis for a 6-month’ period.

### Follow up and re-entry

**Fig. 8:**
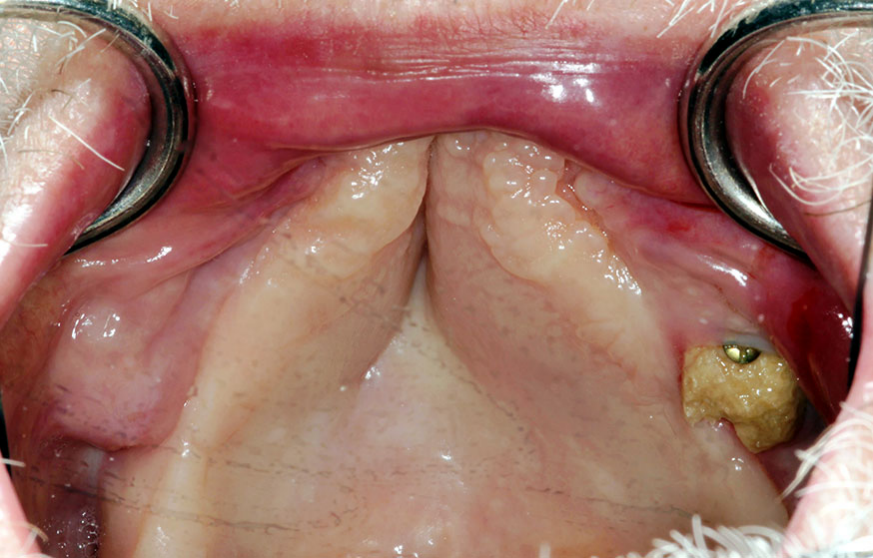
Clinical view: Exposed part of bonebuilder® upper left side six weeks after augmentation and before decortication

The first weeks passed by uneventfully. The bone block made of mineralized collagen gives a stable, osteoconductive lead structure for revascularization and osteoblastic cell migration. Approximately 6 weeks later, an exposed bone was apparent in regio 27 (Fig. 8). Usually, one has to remove the block in such a situation.

Rigid osseous fixation and soft tissue coverage are essential to make treatment successful. The firm, fixed palatal mucosa is clinched in the middle of the palate. Skillful soft tissue management, e.g. elongation, is necessary to cover the palatal area (split-flap). In this case, the mucoperiosteal flap or the thick palatal tissue turned from the middle to the lateral side. The absence of stress in the flap’s joint areas is quite essential (Krasny et al. 2015). To realize this, stabile sutures like mattress sutures are helpful. A few months after augmentation, the attached gingiva showed again in the palatal area.

**Fig. 9:**
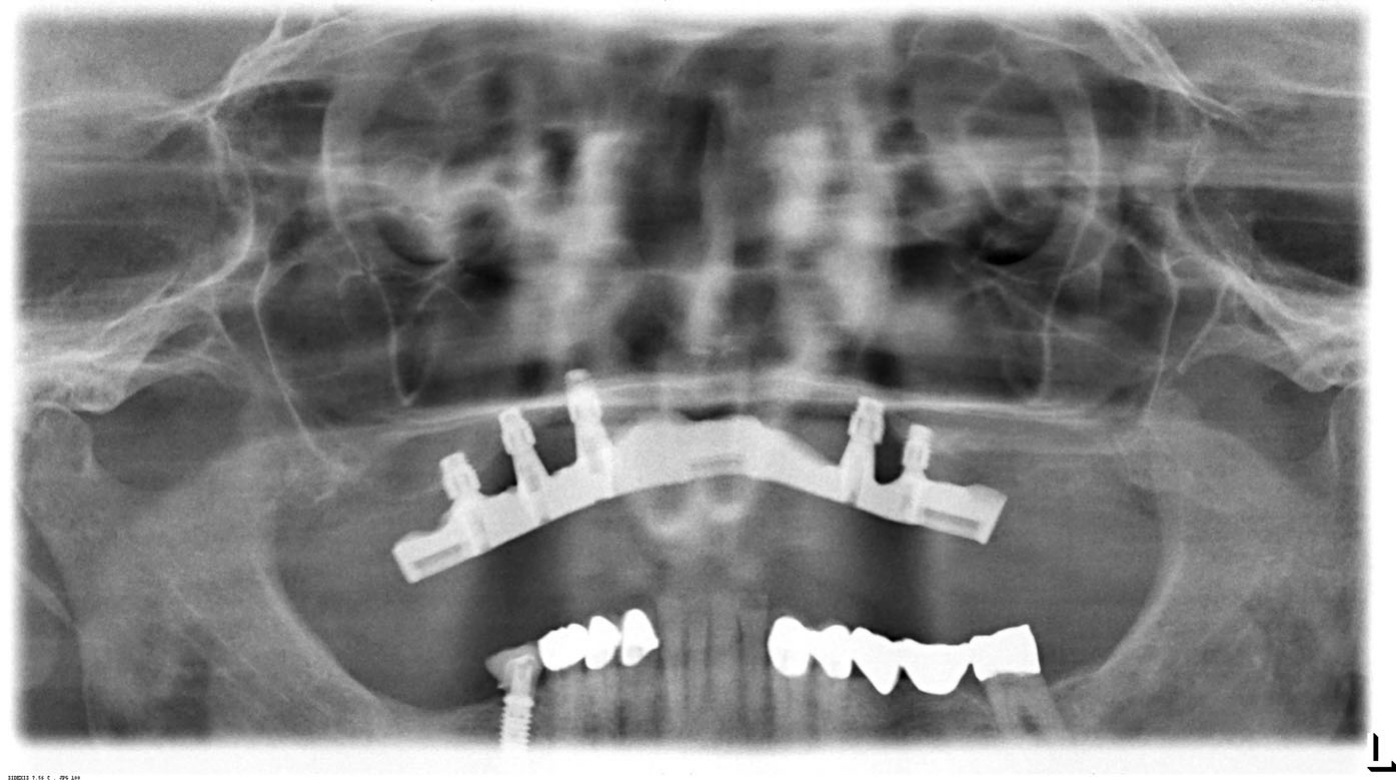
OPT after implant-loading by CAD-CAM- milled bar construction for cover-denture

**Fig. 10:**
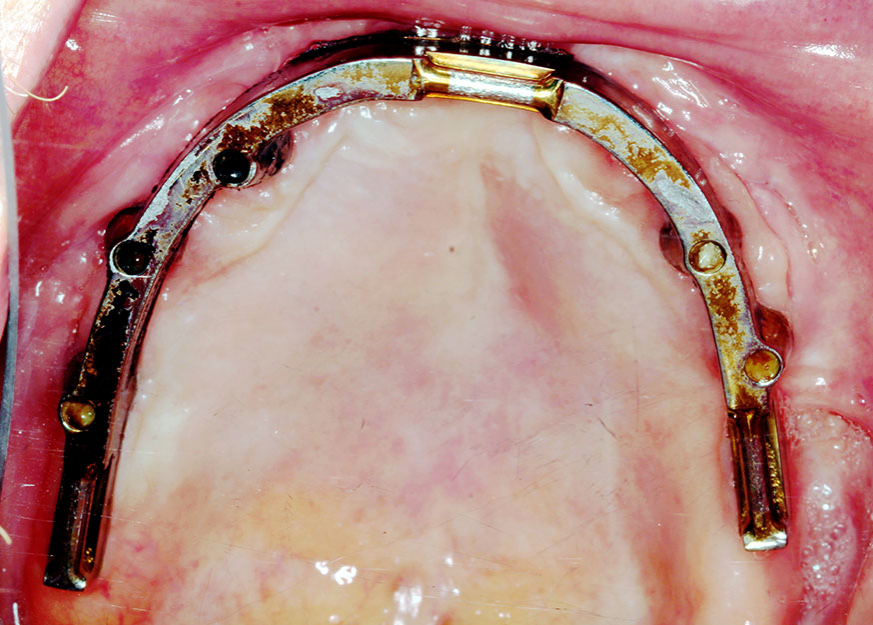
Clinical view: CAD-CAM-milled bar construction for cover-denture prosthetics

We installed six cone morse implants (4.2mm × 7.5 mm; SICmax, SIC-Invent AG, CH-Basel). Three implants each were placed in both sides of the posterior maxilla using a drill template 6 months later. The crestal micro thread is suitable for D2-D4 bone density and compresses the implant into the reconstructed bone. In combination with augmentative, the authors recommend the macrostructure. We lost one implant in that region during implant integration even though it was safely fixed into the remaining bone; it got lost because the bone block was altered by connective tissue. We made a fitted bar abutment supported CAD/CAM-overdenture (Figs. 9 and 10). Six months after implantation, the patient was rehabilitated prosthetically with implant-supported bar construction and cover denture. The index-score of the papilla (Jemt 1997), which was measured every 6 months was stable for 3 years and showed less than a half of the average papilla height. Bleeding on probing was registered according to a modified version of Löe and Sillness (1963) every 6 months. They defined the peri-implant mucosa status as 0: Normal peri-implant mucosa; 1: Bleeding on superficial probing, and 2: Spontaneous bleeding. In the case presented, the patient had normal peri-implant mucosa (Score 0).

### Resorption rates of different augmentation techniques—supplementary case series

We examined 30 patients (24–77 years; mean: 49.7; m: 11, f: 19) 3 years to compare the resorption and the long-term volume stability of allogeneic blocks with other augmentation methods. The ethics committee of the University of Münster approved the study (No. 2020-637-fS).

Vertical and horizontal bone deficits indicated bone augmentation for all patients. All grafts had a healing time of 6 months. 15 patients received allogeneic bone blocks (C + TBA bone blocks, maxgraft, Botiss, Berlin, Germany) as described above. 11 patients were augmented using the shell technique (maxgraft-cortico CHB; Botiss, Berlin, Germany). Nine patients were treated from the iliac crest using cortico-cancellous autologous blocks. The augmented area was measured using CBCT in the XYZ axis (Kavo-3D-Exam, Biberach, Germany; KaVoExamVision software; Version 1.9.3.12) with exposure factors of 120 kV and 36.12 mAs with a 0.1-mm reconstruction interval). The initial volume was measured, the situation after 6 months before implantation and 6 months after implantation was detected. Up to 3 years, we examined postoperatively the bucco-oral, vertical and mesiodistal dimensions. After the augmentation, we used osteosynthesis screws, anatomical landmarks, and, later, the dental implants as orientation.

### Statistical analysis

We tested on both sides and used a significance level of 5%. There was no alpha adjustment for multiple testing. The results are descriptive. IBM SPSS Statistics 25 (SPSS Inc. at IBM Company, Chicago, IL) was used to perform the statistics. Medistat GmbH, Kiel, Germany carried out the statistics.

## Results

Augmentation pre-and post-operative. In all cases of all three groups, a successful augmentation was detected postoperatively. The values preoperatively and 6 months postoperatively were included in the assessment to assess a positive augmentation.

### The course of the bone heights

Table 1 and Diagram 1 show the measurement progression of the bone heights. We recorded vertically, buccolingually, and mesiodistally. The initial value corresponds to the situation before the operation. The second measurement was carried out 6 months after the bone formation and the expected completion of the incorporation. In all cases, one can speak of a good bone structure. Even 1 year after bone augmentation (measurement after 12 months), the bone heights were stable.

**Table 1:** Bone-Resorption after augmentation with Bonebuilder, cortico-plate and hipgraft: Detection [N] of vertical, bucco-lingual, and mesio-distal dimensions [mm; mean; SD] after initial augmentation and 6 monthly follow-up’s for 3 years

Group		N	Mean	SD	Minimum	Maximum	Percentile		
							25	50. (median)	75
Bonebuilder									
Vertical	Augmentation: diff. 6 month–preop [mm]	42	5.73	3.50	1.41	16.85	2.53	5.52	7.99
	Resorption: diff. 12–6 month postop [mm]	32	− 1.03	1.54	− 4.26	1.79	− 1.99	− 0.92	− 0.16
	Resorption: diff. 24–6 month postop [mm]	25	− 2.14	2.20	− 6.11	1.03	− 3.98	− 1.91	− 0.38
	Resorption: diff. 36–6 month postop [mm]	9	− 3.83	1.98	− 6.68	− 0.27	− 5.78	− 3.22	− 3.05
Bucco-lingual	Augmentation: diff. 6 month–preop [mm]	42	5.52	3.48	0.17	14.83	2.62	4.90	7.74
	Resorption: diff. 12–6 month postop [mm]	32	− 0.96	2.25	− 7.33	3.61	− 1.77	− 0.42	0.50
	Resorption: diff. 24–6 month postop [mm]	25	2.03	2.41	− 7.52	3.19	− 3.27	− 2.07	− 0.44
	Resorption: diff. 36–6 month postop [mm]	9	− 4.64	2.42	− 7.87	− 1.52	− 6.86	− 4.38	− 2.00
Mesio-distal	Augmentation: diff. 6 month–preop [mm]	42	2.81	2.36	0.18	8.83	0.95	2.02	4.33
	resorption: diff. 12–6 month postop [mm]	32	− 1.25	2.49	− 6.19	4.94	− 2.43	− 1.11	− 0.09
	Resorption: diff. 24–6 month postop [mm]	25	− 3.51	4.15	− 10.30	2.46	− 6.82	− 2.04	− 0.28
	Resorption: diff. 36–6 month postop [mm]	9	− 4.56	2.68	− 8.12	− 2.49	− 8.12	− 3.08	− 2.49
Cortico-plate (tibia)									
Vertical	Augmentation: diff. 6 month–preop [mm]	13	5.21	3.71	0.50	12.64	2.58	4.59	6.54
	resorption: diff. 12–6 month postop [mm]	11	− 1.71	1.60	− 4.53	0.86	− 3.43	− 1.13	− 0.65
	Resorption: diff. 24–6 month postop [mm]	5	− 2.42	2.83	− 7.01	0.16	− 4.89	− 2.05	− 0.13
	Resorption: diff. 36–6 month postop [mm]	2	− 5.51	3.80	− 8.20	− 2.82	− 6.15	− 5.51	− 2.63
Bucco-lingual	Augmentation: diff. 6 month–preop [mm]	13	7.98	3.74	0.22	14.00	5.97	7.15	11.14
	Resorption: diff. 12–6 month postop [mm]	11	− 0.34	1.33	− 2.84	1.61	− 1.28	− 0.44	0.79
	Resorption: diff. 24–6 month postop [mm]	5	− 0.92	1.94	− 4.13	0.96	− 2.58	− 0.40	0.49
	Resorption: diff. 36–6 month postop [mm]	2	− 4.31	2.09	− 5.79	− 2.83	− 4.34	− 4.31	− 2.22
Mesio-distal	Augmentation: diff. 6 month–preop [mm]	13	3.62	4.25	0.20	15.13	0.50	2.30	5.34
	Resorption: diff. 12–6 month postop [mm]	11	− 0.95	3.22	− 7.56	3.46	− 1.00	− 0.13	0.60
	Resorption: diff. 24–6 month postop [mm]	5	− 0.72	2.51	− 4.48	2.05	− 2.80	− 1.11	1.56
	Resorption: diff. 36–6 month postop [mm]	2	− 2.83	6.86	− 7.68	2.02	− 5.76	− 2.83	1.24
Hipgraft									
Vertical	Augmentation: diff. 6 month–preop [mm]	20	5.47	3.52	1.49	13.26	2.52	4.80	6.84
	resorption: diff. 12–6 month postop [mm]	11	− 0.77	0.75	− 1.80	0.43	− 1.29	− 0.87	− 0.04
	Resorption: diff. 24–6 month postop [mm]	14	− 0.67	1.61	− 3.56	3.69	− 1.83	− 0.62	− 0.08
	Resorption: diff. 36–6 month postop [mm]	0							
Bucco-lingual	Augmentation: diff. 6 month–preop [mm]	22	5.27	3.79	1.40	14.45	2.70	4.22	6.41
	Resorption: diff. 12–6 month postop [mm]	13	− 0.82	3.60	− 9.14	4.40	− 2.84	0.25	1.30
	Resorption: diff. 24–6 month postop [mm]	16	− 0.35	3.72	− 9.13	6.41	− 1.42	− 0.34	1.25
	Resorption: diff. 36–6 month postop [mm]	2	0.21	0.70	− 0.29	0.70	− 0.22	0.21	− 0.01
Mesio-distal	Augmentation: diff. 6 month—preop [mm]	21	3.63	3.22	0.35	12.51	1.10	3.16	4.36
	Resorption: diff. 12–6 month postop [mm]	12	− 2.26	3.73	− 11.43	4.10	− 3.69	− 1.40	− 0.35
	Resorption: diff. 24–6 month postop [mm]	15	− 2.70	3.71	− 10.96	2.80	− 4.19	− 2.70	0.60
	Resorption: diff. 36–6 month postop [mm]	1	1.90	− 5.60	1.90	1.90			

## Discussion

The author uses CAD/CAM-made, allogeneic blocks and individually manufactured for defects for 15 years (Nilius et al. 2019). The efficacy of processed allografts is comparable to autogenic/autologous bone transplants. However, processed allografts belong to bone substitutes, so comparing other xenogeneic or synthetical origin substitutes is reasonable. Animal studies are limited for allografts because human processed bone substitutes in animals are classified as xenogeneic transplant (usually immunological reactions caused by remaining collagen). (Rothamel et al. 2008). A study by Schlee et al. (2013) compares different bone substitutes. There is no significant difference in the rate of bone formation between allogeneic material (35,4 ± 2,8%) and autogenous bone (42,7% ± 2,1%) in maxillary sinus lift.

In comparison to other bone replacement materials, both variants are superior to a bovine demineralized bone matrix (24,9 ± 5,67%). Compared with a biphasic synthetic bone substitute (30,3 ± 2,2%), autogenous bone also showed higher bone formation rates. The superiority of mineralized allografts over deproteinized xenogeneic bone matrix finds proof in a study by Froum et al. (2006).

26 to 32 weeks after sinus floor elevation, there were apparent differences in bone formation rates of 28,3% (allografts) compared to 12,4% (xenografts) (Froum et al. 2006). Even the remaining of a non-vitalized bone substitute was with 7,7% versus 33% better in allografts. The team of authors Laino et al. (2014) concluded that because of the absence of extraction morbidity at the bone graft extraction site and less invasiveness, allogeneic blocks should are preferable. In their survey for sandwich-osteotomy of the lateral lower jaw, there were no notable differences between allogeneic blocks (30,6 ± 3,7%) and autogenous chin blocks (31,47 ± 2,2%). The authors confirm the same results. In comparison between sandwich-osteotomies and onlay augmentations with allogeneic bone segments, a further study showed a higher dehiscence rate in onlay-osteoplasty. One can obtain a two millimeters higher vertical augmentation when the healing period is uneventful and lasts longer (7 months) using onlay-plasty. Other authors confirm these positive results (Vastardis and Yukna 2006; Tudor et al. 2008; Tolstunov and Chi 2011).

For a risk assessment of mineralized processed allografts, a distinction is a need. High standards, which producers must comply with, provide security for the patient and must include the treatment process and the original harvested material. Industrial processing of the material aims to eliminate allergenic and infective parts. Different chemical techniques are used, such as peracetic-acid–ethanol-treatment, thermal disinfection (elimination of potentially contagious agents), lyophilization, osmotic treatment with saline solution, treatment with acetone, and oxygen (elimination of cellular components and fats), and gamma-sterilization. Due to the processing, risks (i.e., the transmission of infection and antigenicity) reduces significantly. Fretwurst et al. (2014) sees no similar immunological reaction using allografts but found isolated cell residues and DNA-parts within the matrix structure of different mineralized, decellularized allografts in block shape. One of the criteria for the success of the treatment protocol and a lower risk of complication is tension-free closure of the wound by “loop” or “pulley” vertical mattress suture]. Exposed allogenic areas have to ablated spaciously.

The trabecular structure of the cancellous allogenic bone allows comparatively fast revascularization. Today non-absorbable membranes 100% made of dense polytetrafluoroethylene (PTFE) are available. Furthermore, we recommend using titan reinforced PTFE-membranes, which has advantages for further implant-supported prosthetic planning. Because of the small pore size, this membrane is an efficient barrier against cellular penetration and reduces wound dehiscence risk. Long-time exposition of the membrane is possible. It is not comparable to fully developed keratinized soft tissue, but it gives a protective barrier for the bone block.

### Dental implantation

A drill template can be made based on the block planning data (3D-bonebuilder). However, bone resorption after 6 months of healing and resilience is unknown. Digital implant planning was done 6 months after insertion based on a CBCT by (Smop, Swissmeda, CH-Baar). The visualization of the bone was not ideal because of the incomplete vascularization. After coverage by a radio-opaque titan-mesh-membrane or a thin layer of radio-opaque bone-substitute in advance, allogeneic transplants’ visibility is better (Fig. 7). Alternatively, setting the Hounsfield units at 200–400 HU range, differentiation of the reconstructed bone is relatively visible.

The implant positioning was challenging. The bone blocks have an average height of one centimeter. Usually, there is an attempt to insert the implants while also using the local bone underneath the augmented area. Because of the loose joint between the transplant and the local bone, there is a high risk of lifting the bone block during implant insertion. Moreover, resorption, which is about 10 to 15% (Nilius et al. 2019), is comparable with native bone grafts. The case presented is one part of a case series of 15 bonebuilder [m:6/w:9] operated the last 5 years (not published yet) compared to cortico-plate (maxgraft-cortico-plate™, allogeneic tibia; shell-technique), and autologous (iliac-crest) bone-augmentation. Their estimated bone-loss is based on descriptive statistics (parametric t-test, Cohen-Effect, Mann–Whitney-U-Test) for maxillary bonebuilder as shown in Tables 1 and 2. The short implant of a maximum of 7.5 mm length and guided surgery may be one good alternative since the allogeneic bone covers them in total.

**Table 2:**
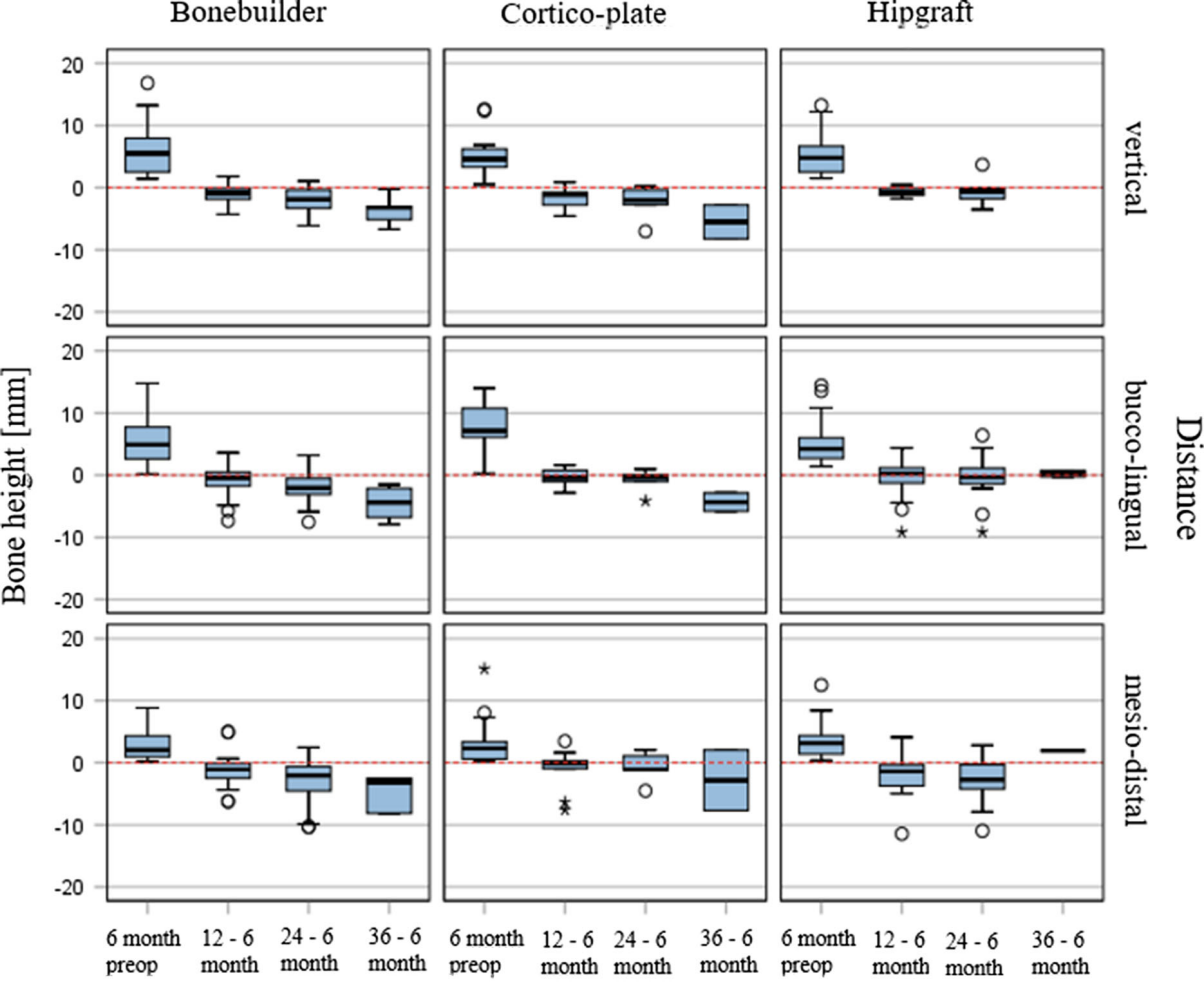
Boxplot-diagram: Resorptionrate after augmentation with Bonebuilder, cortico-plate and hipgraft: Detection [N] of vertical, bucco-lingual, and mesio-distal dimensions [mm; mean; SD] after initial augmentation and 6 monthly follow up fo 3 years

## Conclusion

The case report shows the options for a complete absolute maxillary augmentation using allogenic blocks. The scope of bone-building steps, in this case, is rarely performed so far. For a few years, allogeneic block transplants are CAD/CAM-made individually for the circumscript situation (Schlee and Rothamel 2013). Using allogeneic bone blocks on an individualized basis provides many benefits over autologous bone blocks, e.g., no donor site operation and shorter surgery time. Moreover, the exact fit of milled blocks, mainly dealing with complex defects, justifies the procedure. The success of such therapy depends on many factors. It is not just the osseous fixation of the allogeneic transplant that is important but also covering soft tissue. During the first surgery, it is an expansion and tensionless adaption of mucogingival reserves with the support of a membrane of the augmentation site. The secondary step is a thickening and optimizing of the implant-surrounding soft-tissue structures.

Furthermore, the chosen implant system plays a significant role. Short implants are suitable if they have a special macrostructure. Crestal micro threads, for example, are quite useful for D2–D4 bone density and compress the implant even into the reconstructed bone; cutting threads combined with reduced implant bodies may a possible option.

## Abbreviations

CBCT: Conebeam-computer tomography
DVT: Digital volume tomography
D1–D4: Bone density
D1: Hard bone
D4: Soft bone
HU: Hounsfield unit
MPBA: Mineralized processed human bone
OPT: Orthopantomography
